# First person – Keegan Bush

**DOI:** 10.1242/bio.058536

**Published:** 2021-01-28

## Abstract

First Person is a series of interviews with the first authors of a selection of papers published in Biology Open, helping early-career researchers promote themselves alongside their papers. Keegan Bush is first author on ‘*Drosophila* model of anti-retroviral therapy induced peripheral neuropathy and nociceptive hypersensitivity’, published in BiO. Keegan is a PhD Student in the lab of Yogesh Wairkar and Shao-Jun Tang in the departments of Neurology and Neuroscience, and Cell Biology and Anatomy, at Galveston University, Texas, USA, investigating how anti-retroviral drugs cause nociceptive hypersensitivity in people living with HIV.


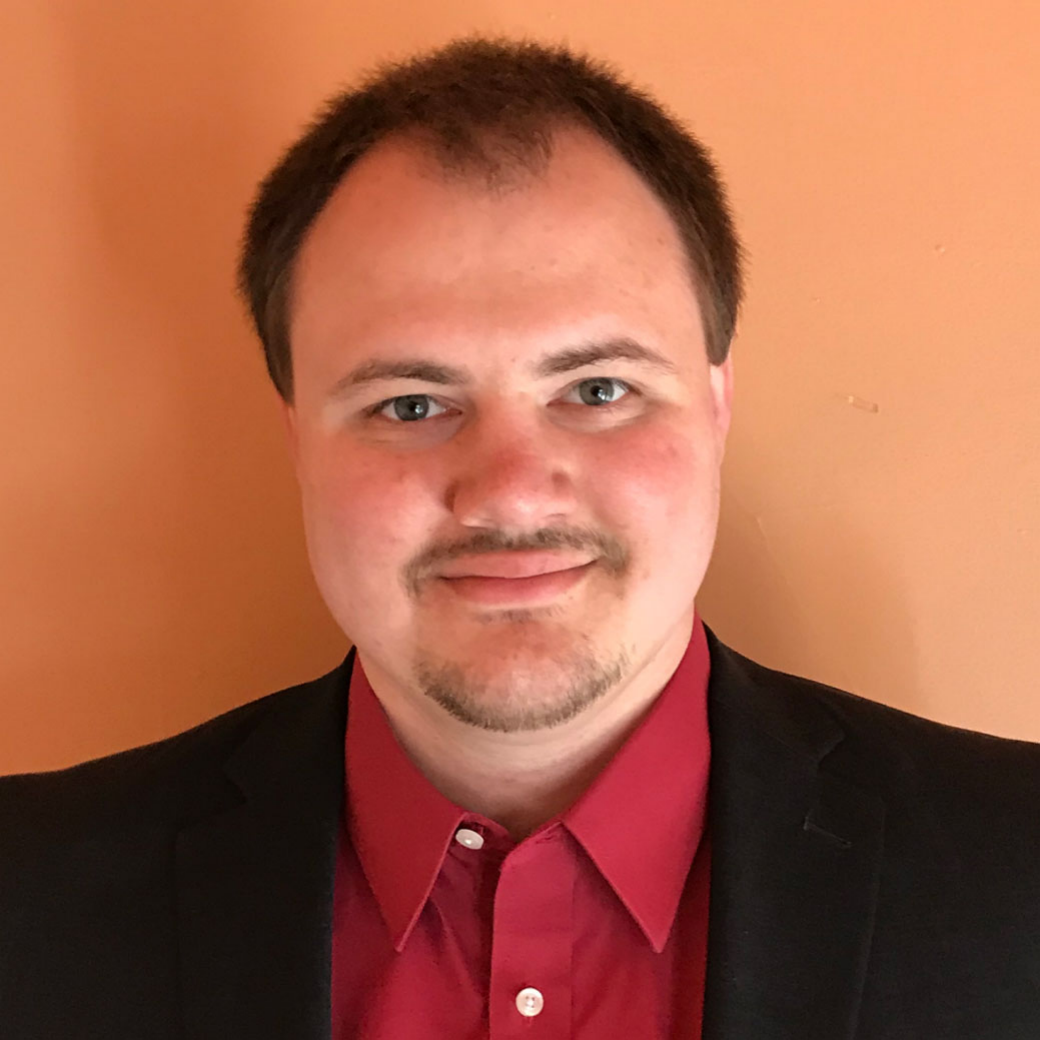


**Keegan Bush**

**What is your scientific background and the general focus of your lab?**

Dr Wairkar's lab is interested in understanding neurodegenerative diseases from the perspective of synapse loss. Virtually all neurodegenerative conditions lead to some sort of loss of synapses, which are the main conduits of communication between neurons and their targets. Dr Tang's lab is interested in understanding nociception underlying HIV-disease. Therefore, these two labs were a perfect home for me to understand how peripheral neuropathies and nociceptive hypersensitivity are caused by the anti-retroviral therapies that are commonly given to people surviving with HIV.

**How would you explain the main findings of your paper to non-scientific family and friends?**

HIV disease used to be one of the most lethal diseases several decades ago. The advent of drugs that stop the replication of this virus has been absolutely a boon for people who contract the disease. In fact, many people who take these drugs, commonly referred to as anti-retrovirals, can live relatively normal lifespans with the HIV disease. The patients living with HIV commonly exhibit pain and numbness symptoms in their extremities like fingers and toes, and recent studies have indicated that the anti-retroviral drugs can themselves cause destruction of nerves that supply these extremities, leading to the painful symptoms in these patients. How the anti-retroviral drugs cause these symptoms is not known. My work provides a powerful model to understand how these drugs may cause the symptoms and also provides hints into possibilities of how the peripheral neurodegeneration might be happening at a molecular level.

“My work … hints into possibilities of how the peripheral neurodegeneration might be happening at a molecular level.”

**What are the potential implications of these results for your field of research?**

My work provides a powerful model in which to study how anti-retroviral drugs lead to progressive degeneration of peripheral nerves. Currently, we are investigating these phenomena in mice, which have been a powerful model to understand causes of pain. If successful, my work would be a stepping stone to further understanding the degeneration of nerves due to the use of the anti-retroviral drugs and could potentially provide possible ways to counteract the problem.

“… I have been surprised about how accurately the data from *Drosophila* has so far been replicated in the mouse model …”

**What has surprised you the most while conducting your research?**

Almost all the data in this paper was acquired using the *Drosophila* model. While the current work that I am performing is not yet published, I have been surprised about how accurately the data from *Drosophila* has so far been replicated in the mouse model and the mechanisms of nociception also seem to be very well conserved. These are good indications for the future prospects of this project.

**Figure:**
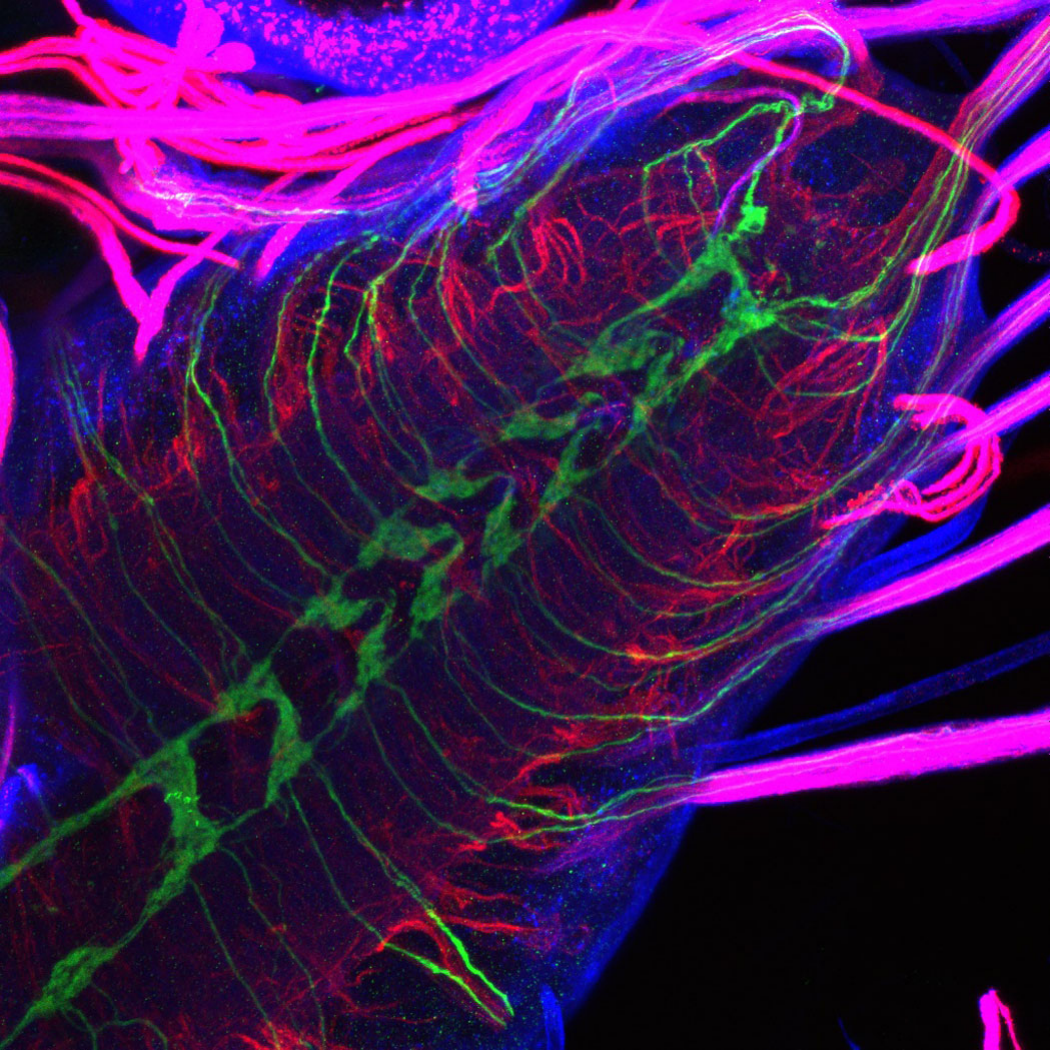
**Image of the ventral nerve cord (insect spinal cord analogue) of a *Drosophila* larva.** The green is GFP labelling of the larva sensory neurons. The red is futsch staining which labels microtubules. The blue is HRP staining of general neuronal membrane.

**What, in your opinion, are some of the greatest achievements in your field and how has this influenced your research?**

Translational research utilizing *Drosophila* is often viewed with skepticism due to the difference in their genomes with the vertebrates. But many studies have shown the utility of *Drosophila* for translational research and so far, my data support that idea. Recent work that utilized *Drosophila* to examine and understand the chemotherapeutic treatments and nociception from the labs of Drs Williams, Galko, Neely, DiAntonio and Zhai was one of my inspirations for this project. I would say that the relative scarcity of the models to study anti-retroviral therapy was an inspiration too, because so much can be learnt from *Drosophila* due to its unparalleled genetics and because of decades of work in nociception in *Drosophila*. I thought that this was a great opportunity to make a meaningful contribution.

**What changes do you think could improve the professional lives of early-career scientists?**

Many projects investigated by early-career scientists are focused on a single avenue of research. This is a result of their topic typically being constricted to procedures available in their labs. The diversification of your knowledge base greatly affects approaches to experimental design and its lack may hinder effective research strategies. Some ways to remedy this would be to provide more access to observation of techniques utilized in other labs as part of a graduate student course, or to increase the prevalence of the co-mentorship of students between multiple labs. This would not only bring these alternate techniques to mind in designing experiments, but also provide greater value to these scientists in the eyes of any future lab they may later join.

**What's next for you?**

Once my research at UTMB is at a stopping point and I graduate, I plan to move to another lab in a postdoctoral position. I plan to remain in the field of neurodegeneration, but I would like to expand my knowledge base to other neurodegenerative diseases, which may have a different origin and different challenges. My long-term goal is to remain in research where there is a high possibility for translating the bench research to help patients with neurodegenerative conditions, which would have a positive impact on society.
